# Elastin Insufficiency Predisposes Mice to Impaired Glucose Metabolism

**DOI:** 10.4172/1747-0862.1000129

**Published:** 2014-09-30

**Authors:** Antea J DeMarsilis, Tezin A Walji, Justine A Maedeker, Kellie V Stoka, Beth A Kozel, Robert P Mecham, Jessica E Wagenseil, Clarissa S Craft

**Affiliations:** 1 Department of Cell Biology & Physiology, Washington University in St. Louis, MO, USA; 2 Department of Mechanical Engineering and Materials Science, Washington University in St. Louis, MO, USA; 3 Department of Pediatrics,, Washington University in St. Louis, MO, USA

**Keywords:** Williams syndrome, Elastin, Elastic fiber, Apolipoprotein E, Cardiovascular disease, Diabetes, Adipose tissue

## Abstract

Williams-Beuren syndrome is the consequence of a large contiguous-gene deletion on the seventh human chromosome that includes the elastin gene. Elastin is an extracellular matrix protein responsible for the cardiovascular abnormalities associated with Williams’s syndrome, including hypertension and aortic stenosis. A high percentage of individuals with Williams’s syndrome also have impaired glucose tolerance, independent of traditional risk factors for diabetes. Here, we show that murine adipose tissue does assemble elastic fibers; however, isolated elastin insufficiency (*Eln*^+/−^) in mice does not independently influence glucose metabolism or tissue lipid accumulation. Similarly, isolated ApoE deficiency (*ApoE*^−/−^), a model of hyperlipidemia and atherosclerosis, does not impair insulin sensitivity. However, *Eln*^+/−^; *ApoE*^−/−^ double mutant mice exhibit notable hyperglycemia, adipocyte hypertrophy, inflammation of adipose tissue, and ectopic lipid accumulation in liver tissue. Further, *Eln*^+/−^; *ApoE*^−/−^ mutants have significant impairment of insulin sensitivity by insulin tolerance testing, independent of body weight or diet, suggesting that elastin insufficiency predisposes to metabolic disease in susceptible individuals.

## Introduction

Elastic fibers confer elasticity and resilience to many tissues, including arterial walls. Elastic fiber assembly occurs through the cross-linking of tropoelastin by lysyl oxidase, followed by deposition of these elastin aggregates onto fibrillin-rich microfibrils in the extracellular milieu [1]. Degradation of elastic fibers is facilitated through expression of serine or matrix metalloproteinases (MMPs) having elastase activity. Alteration to elastic fibers, whether by mechanisms of impaired elastic fiber assembly or increased degradation, has been implicated in the development of Williams-Beuren syndrome (Williams syndrome, WS) and cardiovascular disease, including hypertension, supravalvular aortic stenosis (SVAS), and atherosclerosis [2–4].

WS is a genomic disorder presenting with cardiovascular, endocrine, and developmental abnormalities in 1/7,500–1/12,000 individuals [5,6]. WS is caused by the deletion of 26–28 genes, including elastin (ELN), at chromosome position 7q11.23. Elastin insufficiency is known to produce many of the characteristic cardiovascular abnormalities in WS, including focal arterial stenosis, hypertension, and increased vascular stiffness [7–9]. Clinical studies demonstrate that individuals with WS also represent a group with one of the highest frequencies of impaired glucose tolerance of any human population [5,10,11]. In one study, 75% of adults with WS met criteria for diabetes or pre-diabetes, with glucose dysregulation observed independently of BMI and other traditional risk factors for diabetes [11]. Because of its high clinical prevalence, it has been suggested that abnormal glucose metabolism in WS is genetically predisposed. Within the WS deletion are genes such as STX-1A and MLXIPL that have been shown to be important regulators for glucose metabolism and their hemizygosity, and are therefore thought to contribute to the impaired glucose metabolism associated with WS [11].

The extracellular matrix (ECM) is known to play a critical role in chemical and mechanical regulation of adipose tissue function [12], and there is precedence for microfibril-elastic fiber involvement in metabolism. For example, fibrillin-1 mutations, in humans with Marfan syndrome, are often associated with severe fat hypoplasia [13]. We also recently showed that MAGP1 – a microfibril-associated protein – has a regulatory role in energy utilization through its interaction with TGFβ, and mice lacking MAGP1 have reduced energy expenditure, accumulate excess fat mass and develop insulin resistance [14]. Further, Blaise et al. recently showed that elastin-derived peptides (EDPs) cause insulin resistance in skeletal muscle, liver and adipose tissue [15]. To further our understanding of ECM contributions to energy metabolism and the etiology of impaired glucose tolerance in Williams syndrome, adipose tissue structure and glucose regulation were evaluated in mice with isolated elastin hemizygosity (*Eln*^+/−^).

## Materials and Methods

### Animals and diets

All animals were treated in accordance with animal protocols approved by the Animal Studies Committee at Washington University. Generation and genotyping of *Eln*^+/−^ mice on a majority c57Bl/6 background has been previously described []. Generation of *Eln*^+/−^; *ApoE*^−/−^ (double mutant) mice is as follows: Female, B6.129P2-Apoetm1Unc/J,^−/−^ (*ApoE*^−/−^) mice were purchased from The Jackson Laboratory (Bar Harbour, ME). *ApoE*^−/−^ mice were then crossed with *Eln*^+/−^ mice. Study mice were littermates from the F3 through F5 generations that resulted from these crosses. Mice were housed in a pathogen-free animal facility and were maintained under a 12h light: 12h darkness cycle. All study mice were male and on c57 strain background. Animals were fed standard, high-fat diet (HFD), or Western diet and water ad libitum. For HFD studies, mice were placed on HFD or control chow for 16 weeks (D12492 (HFD, 60% fat) or D12450B (control, 10% fat); Research Diets, Inc., New Brunswick, NJ). For Western diet studies, mice at 4 weeks of age were placed on control chow or Western diet for 12 weeks (5432 AIN-76A (Western Diet, 20% fat) and 5755 Basal Diet (control, 10% fat); TestDiet, St. Louis, MO).

### Glucose and insulin tolerance testing

For glucose tolerance tests (GTTs), mice were fasted 6 hours before a 1g/kg dextrose injection. For insulin tolerance tests (ITTs), mice were fasted 6 hours before 0.75 units/kg Humulin-R insulin injection (Lilly, Indianapolis, IN). Dextrose and insulin were delivered by intraperitoneal injection. Tail blood glucose concentration was measured at the indicated intervals using Contour strips and meter (Bayer, Whippany, NJ).

### Histology and immunohistology

Adipose and liver tissues were fixed in 10% buffered formalin, dehydrated by an ethanol gradient, and stored in 70% ethanol at 4°C before paraffin embedding. Liver and adipose tissue sections were incubated with hematoxylin and eosin or Masson’s trichrome, stain. Elastin protein deposition was visualized by incubating adipose tissue sections with anti-Elastin antibody, as described previously [17]. Images were captured using an Olympus Nanozoomer 2.0-HT System with NDP.scan 2.5 imaging software. For adipose tissue adipocyte size and number, ImageJ software was used to make measurements in four fields per section.

### Stromal vascular fraction (SVF) isolation and immunofluorescence

Inguinal subcutaneous white adipose tissue (scWAT) fat pads were dissected and minced in phosphate buffered saline (PBS). Minced tissue was digested in 0.32 units/ml Liberase TM (Roche Diagnostics, Mannheim, Germany) at 37°C for 40 minutes with constant agitation. After incubation, the resulting cell suspension was passage through 100 µm filter, centrifuged to separate floating adipocytes from the SVF pellet, and the SVF pellet was treated with Red Blood Cell Lysing Buffer (Sigma Aldrich, Saint Louis, Mo). The resulting cells were cultured in DMEM/F-12 (Life Technologies, Carlsbad, CA) supplemented with 10% fetal bovine serum (FBS) and 100 units penicillin/streptomycin (complete medium). Twenty-one days post-confluence, time for sufficient elastic fiber assembly, the cells and the extracellular matrix (ECM) were fixed in ice-cold methanol, and incubated for 1 hour at RT with primary antibody (rabbit anti-mouse recombinant tropoelastin antibody [17] both 1:100) in “wash” buffer (20mM HEPES (Life Technologies, Carlsbad, CA) containing 150 mM sodium chloride, 0.01% Triton X-100, 2% fish gelatin, and 1% bovine serum albumin (BSA)). Cells were thoroughly washed in buffer solution for 15 minutes at RT and then incubated in secondary antibody solution (goat anti-rabbit IgG 1:1000 in buffer solution and Hoechts nuclear stain 1:10000) for 1 hour at RT. Cells were then washed at RT for 15 minutes in buffer solution before imaging

### RNA preparation and quantitative RT-PCR

Adipose and liver tissues were dissected, frozen in liquid nitrogen, and stored at −80°C. RNA was extracted from tissue using TRIzol reagent (Invitrogen, Grand Island, NY) and RNeasy columns (Qiagen, Valencia, CA). RNA was reverse-transcribed using Applied Biosystems (Grand Island, NY) cDNA-to-RNA reverse transcription kit, and then quantitative PCR (qPCR) was performed using Taqman Universal PCR Master Mix reagent kit (Applied Biosystems) on a ViiA 7 Real-Time PCR System (Applied Biosystems). 36B4 and cyclophilin A were used as internal controls.

### Statistical analysis

Results are reported as mean ± standard error of the mean (SEM). Data was analyzed using GraphPad Prism software (GraphPad Software, La Jolla, CA). Student’s t-tests were used to determine differences between groups. For group comparisons, two-way ANOVA with Bonferroni post-hoc corrections were performed with GraphPad Prism software. Values of P ≤ 0.05 were considered significant.

## Results

### Elastic fiber assembly in adipose tissue and remodeling in obesity

**Figure 1:**
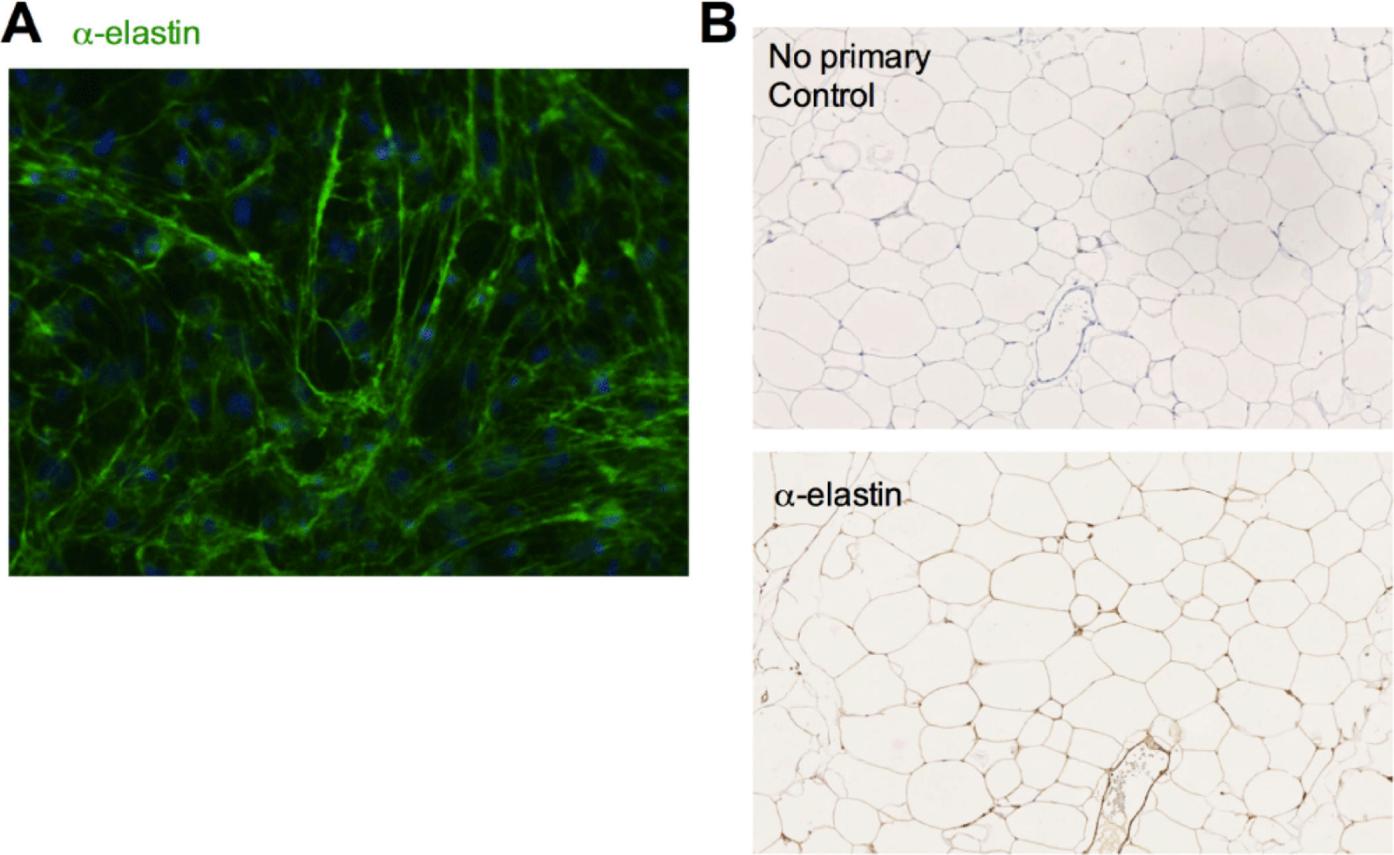
Elastic fibers are present in adipose tissue. A: Elastic fiber (green) assembly and nuclei (blue) assessed by immunofluorescence microscopy of subcutaneous white adipose tissue (scWAT) SVF at 21 days post-confluence with α-elastin and Hoechts stain, respectively. B: Immunohistochemistry with anti-elastin antibody of Eln WT HFD epididymal white adipose tissue (epWAT) sections: hematoxylin stained with no primary (top) or anti-elastin primary antibody (bottom).

While it is established that the collagen network plays a significant role in adipose tissue structure and is remodeled in obese subjects [12], the arrangement and role of other ECM components, including elastic fibers, in adipose tissue is less well-defined. To investigate the presence of elastic fibers in murine adipose tissue, elastin synthesis and deposition were evaluated both *ex vivo* and *in vivo*. Immunofluorescence microscopy with an antibody specific to murine elastin demonstrated that cells in the stromal vascular fraction (SVF) of subcutaneous white adipose tissue (scWAT) express elastin and assemble an elastic fiber matrix when grown in culture (Figure1A). Elastic fibers were also detected by immunohistology throughout intact adipose tissue where they had a pericellular distribution around adipocytes and were associated with blood vessels (Figure 1B).

**Figure 2:**
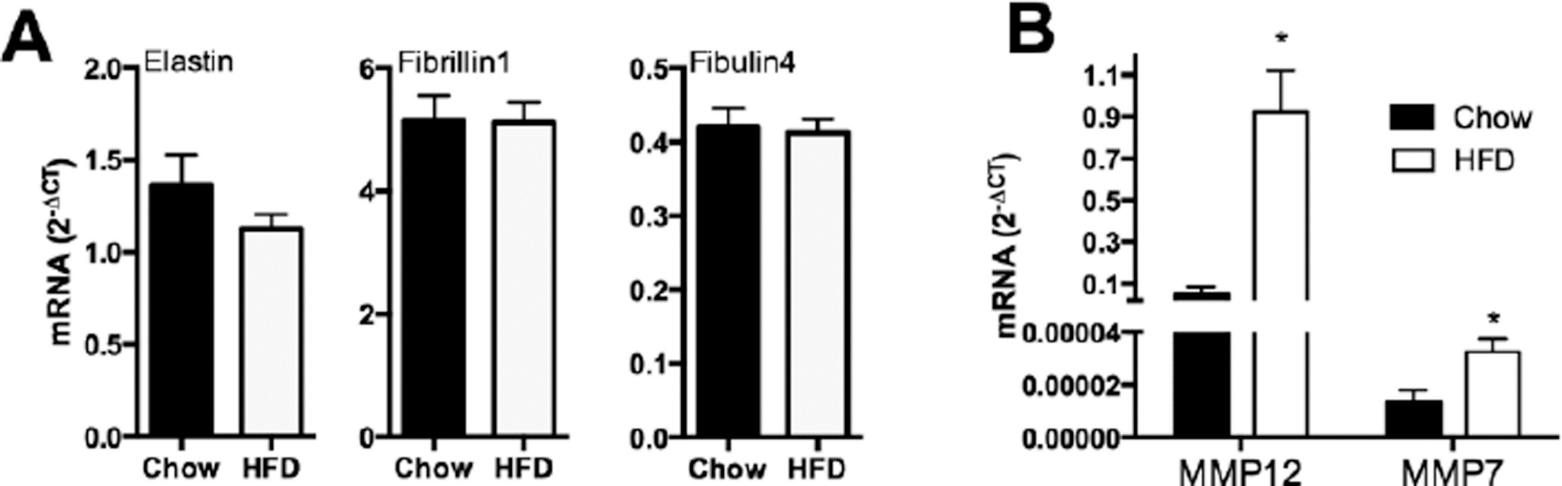
Remodeling of elastic fibers during obesity. A: qPCR expression of elastic fiber microfibril protein-associated genes (*Eln* [elastin], *Fbn*1 [fibrillin-1], *Efemp2* [fibulin-4]) in RNA extracted from epWAT of WT mice fed control chow or HFD (mean ± SEM; n = 6). B: Differential expression of ECM degradation-associated genes in RNA extracted from epWAT of mice fed HFD (*MMP12* [matrix metalloproteinase-12], *MMP7* [matrix metalloproteinase-7], *Elane* [neutrophil elastase]) (mean ± SEM; n = 6). Student’s T-test was used to determine differences between groups, *P≤ 0.05.

To continue investigating the role of elastin in metabolic and obesity pathology, we performed qPCR analysis of key elastic fiber assembly and degradation-associated genes. WT mice fed a high fat diet become obese [14]; however, expression of elastin (*Eln*), fibrillin-1 (*Fbn1*), or fibulin-4 (*Efemp2*) in epididymal white adipose tissue (epWAT) does not change (Figure 2A). If obesity causes remodeling of the fat pad ECM, we should see increased expression of elastases and other matrix remodeling enzymes in this tissue, and an influx of macrophages into fat tissue. Transcript expression of macrophage elastase (*MMP12*) was increased by high-fat feeding in WT mice (Figure 2B). Expression of *MMP7* was also increased to a significant degree, but given the low abundance of *MMP7* mRNA, it is unclear whether this enzyme is physiologically relevant at this stage in adipose tissue.

### Elastin insufficiency alone does not cause impaired glucose tolerance

**Figure 3:**
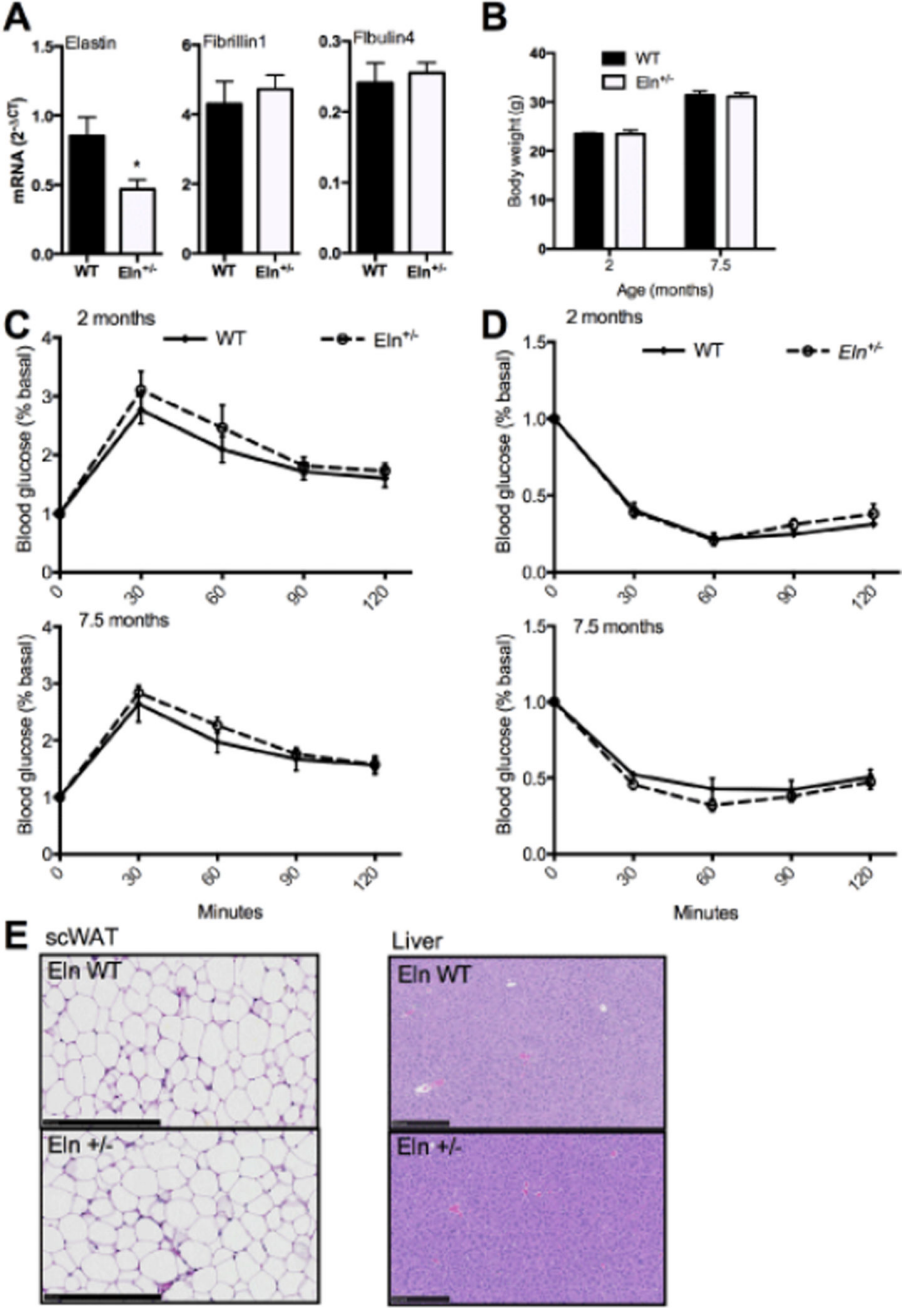
Elastin insufficiency alone does not cause impaired glucose tolerance. A: qPCR expression of elastic fiber microfibril protein-associated genes (*Eln* [elastin], *Fbn1* [fibrillin-1], *Efemp2* [fibulin-4]) in RNA extracted from epWAT of Eln WT and *Eln*^+/−^ mice fed control chow (mean ± SEM; n = 5). B: Body weight of 2-month-old and 7.5-month-old mice (mean ± SEM; n = 4–7). C: GTT results in 2- and 7.5-month-old mice Eln WT and *Eln*^+/−^ mice, following a 6-h fast and 1g/kg dextrose injection (mean ± SEM; n = 4–7). D: ITT results following a 6-h fast and .75 units/kg insulin injection in 2- and 7.5-month-old mice Eln WT and *Eln*^+/−^mice (mean ± SEM; n = 4–6). E: Hematoxylin-eosin staining of scWAT (20×) and liver tissue (10×) in 16-week-old Eln WT and *Eln*^+/−^ mice on control chow (scale bar 250 µm). *P≤ 0.05.

The expected half-normal elastin mRNA level associated with elastin insufficiency in the epWAT of *Eln*^+/−^ mice was confirmed by qPCR analysis (Figure 3A). Importantly, elastin insufficiency, itself, was not associated with decreased expression of other microfibrilar genes (*Fbn*1 and *Efemp*2) (Figure 3A). Body weights are not different between *Eln*^+/−^ and WT groups at 2 or 7.5 months of age (Figure 3B). Glucose tolerance testing (GTT) demonstrated that glucose clearance in *Eln*^+/−^ mice was slightly, but not significantly, reduced relative to WT mice. Insulin tolerance testing (ITT) showed no change in insulin sensitivity between groups (Figure 3C and D). Histological analysis of hematoxylin-eosin staining of scWAT sections showed no evidence of adipocyte hypertrophy or lipid accumulation in liver tissue sections in the *Eln*^+/−^ mice (Figure 3E). Furthermore, similar to a control diet, elastin insufficiency alone had no effect on insulin sensitivity when both WT and *Eln*^+/−^ mice were fed a high fat Western diet (Figure 5D).

### Elastin insufficiency has metabolic consequences in ApoE deficient mice

**Figure 4:**
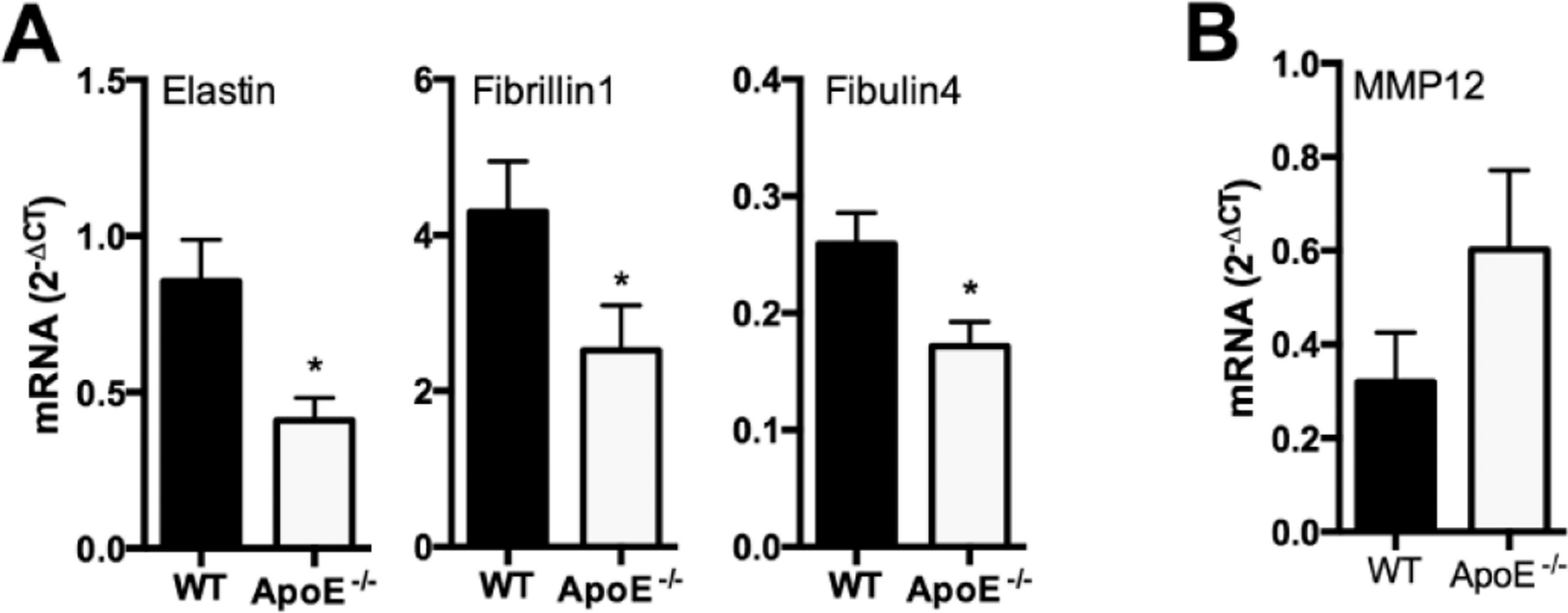
ApoE deficiency alters the expression of elastic fiber-associated genes. A: qPCR expression of elastic fiber microfibril protein-associated genes (*Eln* [elastin], *Fbn1* [fibrillin-1], *Efemp2* [fibulin-4]) in RNA extracted from epWAT of 16-week-old WT and *ApoE*^−/−^ mice fed control chow (mean ± SEM; n = 4–6). B: Differential expression of macrophage elastase-associated gene MMP12 (matrix metalloproteinase-12) in RNA extracted from epWAT of 16-week-old WT and *ApoE*^−/−^ mice on control chow (mean ± SEM; n = 4–6). *P≤ 0.05.

The consequence of elastin insufficiency on glucose regulation was then tested in a second model, one that is associated with cardiovascular and metabolic dysfunction. ApoE is a key regulator of macrophage activity and lipid metabolism. In mice, ApoE deficiency causes the formation of atherosclerotic lesions, hyperlipidemia, and chronic inflammation [18]. Isolated ApoE deficiency alone causes significant changes in elastic fiber-associated gene expression. Specifically, epWAT from *ApoE*^−/−^ mice had significantly reduced transcript levels of *Eln*, Fbn1 and *Efemp2* genes (Figure 4A). *MMP12* (macrophage elastase) was elevated in *ApoE*^−/−^ epWAT, although the change did not reach statistical significance (Figure 4B).

**Figure 5:**
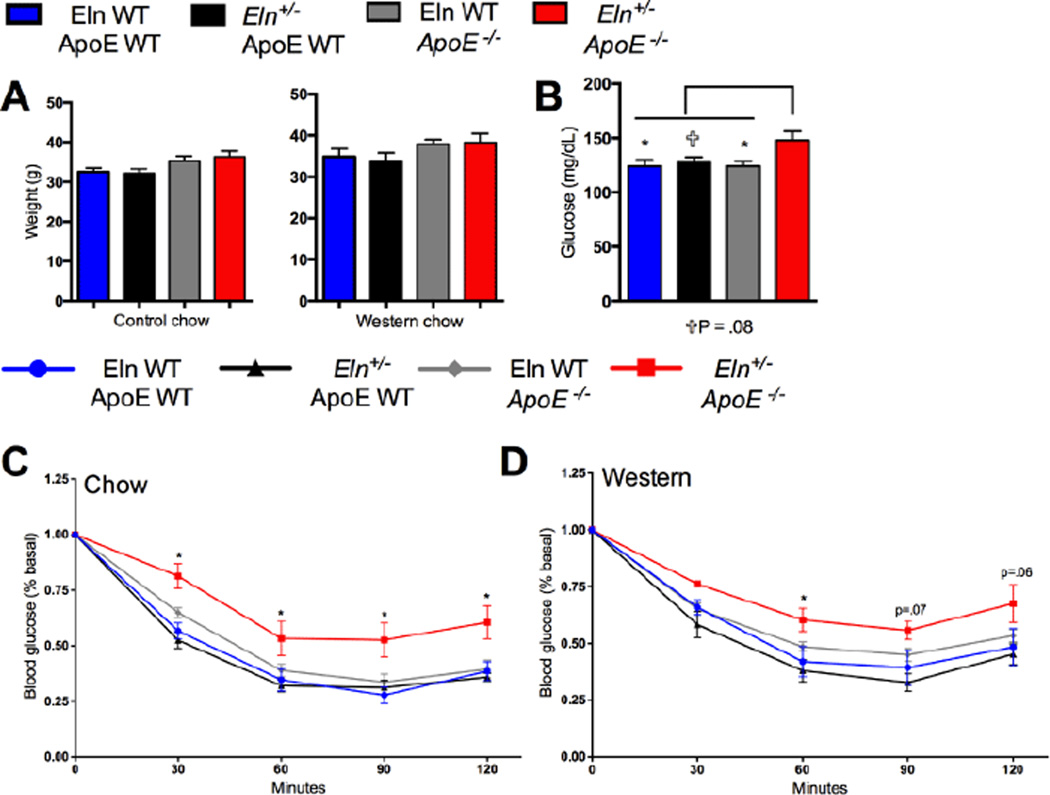
Elastin insufficiency causes metabolic complications in the ApoE-deficient mice. A: Body weight of 16-week-old mice (mean ± SEM; n = 5–18). B: Fed morning glucose measurements of mice on control chow (mean ± SEM; n = 7–19). C: ITT results following a 6-h fast and .75 units/kg insulin injection in Eln WT and *Eln*^+/−^ mice on an ApoE WT or *ApoE*^−/−^ background fed control chow (mean ± SEM; n = 6–19). Significance indicated for two-way ANOVA analysis comparing *Eln*^+/−^;*ApoE*^−/−^ mice to all other groups. D: ITT results following a 6-h fast and 0.75 units/kg insulin injection in Eln WT and *Eln*^+/−^ mice on an ApoE WT or *ApoE*^−/−^ background fed Western diet for 12 weeks compared with control mice (mean ± SEM; n = 6–19) Significance indicated for two-way ANOVA analysis of *Eln*^+/−^;*ApoE*^−/−^ mice compared to Eln WT;ApoE WT mice.^*^P≤ 0.05. ✞P=0.08.

Elastin insufficiency has no consequence on body weight, regardless of diet or *ApoE* genotype (Figure 5A). In control diet fed mice, assessment of blood glucose concentration revealed that neither elastin insufficiency (*Eln*^+/−^) nor ApoE deficiency (*ApoE*^−/−^) alone had any consequence on fed glucose levels. However, elastin insufficiency coupled with ApoE deficiency (*Eln*^+/−^;*ApoE*^−/−^) was associated with hyperglycemia (Figure 5B). Similarly, ITTs demonstrated significant changes in insulin sensitivity among the double mutant mice (*Eln*^+/−^; *ApoE*^−/−^), but not in the single mutants (*Eln*^+/−^ or *ApoE*^−/−^) mice. Specifically, *Eln* +/−; *ApoE* −/− mice have reduced insulin sensitivity (Figure 5C), by two-way ANOVA with Bonferroni post-hoc correction against all other groups.

This finding is maintained in *Eln*^+/−^; *ApoE*^−/−^ mice fed a Western diet (Figure 5D). Analysis by two-way ANOVA (Bonferroni post-hoc) shows significant change in *Eln*^+/−^;ApoE^−/−^ from Eln WT;ApoE WT, though statistical significance is not reached when compared to all groups (*ApoE*^−/−^ mice fed a western diet have slightly reduced insulin senstitivity relative to WT mic*e*). These results demonstrate that elastin insufficiency paired with ApoE deficiency impairs insulin sensitivity, independently of body weight or even diet.

## Discussion

Elastin insufficiency has well-established consequences for cardiovascular disease, and now our findings demonstrate that elastin levels have implications for metabolic health. A greater understanding of the complex pathophysiology of insulin resistance has been achieved in recent years, and there is increasing evidence that the ECM plays an essential role in adipose tissue [19]. Perturbations in adipose tissue ECM alter adipose tissue function [12,14,20–23]. ECM proteins can provide both mechanical and biochemical cues to cells. The collagen network has a mechanical function in adipose tissue whereby it limits adipocyte expansion. However, the presence of fibrosis (increased collagen protein) that occurs in adipose tissue of obese subjects is thought to contribute to metabolic abnormalities [12,20,21]. We recently demonstrated that the extracellular matrix protein MAGP1 influences adipose tissue homeostasis by regulating the capacity for biochemical signaling molecules to reach their target cell [].

Alkhouli et al. showed that an elastin matrix is distributed alongside adipocytes in human subcutaneous white adipose tissue and acknowledged the potential for an important mechanical role in adipose tissue [19]. Other investigators have suggested a role for elastin in influencing metabolism directly. Blaise et al. previously implicated elastin derived peptides (EDPs) in the development of insulin resistance [15]. Further, individuals with WS with half-normal elastin levels are at higher risk of developing impaired glucose tolerance [5,10,11]. We explored how elastin levels influence metabolism using mice engineered to express only one copy of elastin (*Eln*^+/−^). We show that *Eln*^+/−^ mice, relative to WT controls, have normal glucose tolerance and insulin sensitivity. Further, insulin sensitivity following metabolic challenge with Western diet feeding was unaffected by elastin insufficiency. These results show that elastin loss-of-function mutations, by themselves, are not causative for the impaired glucose tolerance in the Williams syndrome population.

In examining the various mouse lines generated for this study, we were surprised to find significantly impaired glucose regulation in animals deficient in ApoE and haploinsufficient for elastin (*Eln*^+/−^;*ApoE*^−/−^). This was not the case in either of the single mutants. The double mutant mice exhibited hyperglycemia on control chow compared to WT mice, mice with elastin insufficiency alone, or mice with ApoE deficiency alone. At a cellular level, we observed significant WAT adipocyte hypertrophy and lipid accumulation in livers from *Eln* +/−;*ApoE* −/− mice, compared to ApoE deficient mice without predisposition of elastin insufficiency (*Eln* WT;*ApoE*−/−). Our analysis also showed that inflammation in the WAT is increased in *Eln* +/−;*ApoE* −/− mice compared *Eln* WT;*ApoE* −/−. Adipocyte expansion, WAT inflammation and ectopic lipid accumulation are highly associated with the pathogenesis of metabolic syndrome [24–28], and are likely critical to the observed insulin resistance phenotype in *Eln* +/−;*ApoE* −/− mice.

Approximately half of the individuals with essential hypertension are insulin resistant [29]; however, diabetes is typically considered to be the risk factor for cardiovascular disease. Our finding that elastin insufficiency has metabolic consequences in an ApoE-deficient background, but not in a diet-induced obese model is intriguing. In contrast to Western diet fed WT mice, *ApoE* −/− mice develop cardiovascular disease (hypertension). This raises the possibility that severe hypertension or cardiovascular disease, as would be predicted by a dual state of elastin insufficiency and ApoE deficiency, can cause predisposition to metabolic dysfunction. This could be easily tested by treatment of *Eln* +/−;*ApoE* −/− mice with anti-hypertensive drugs to see if insulin-resistance can be prevented or reversed.

In this manuscript, the elastin network is shown to be present in murine adipose tissue, and our results implicate elastin insufficiency as a susceptibility factor to metabolic disease in mice. These findings could be of clinical significance as they suggest a causative link between cardiovascular complications and altered glucose metabolism, as well as, introduce the possibility that elastin insufficiency can indirectly contribute to the impaired glucose tolerance associated with Williams syndrome.

## Figures and Tables

**Figure 6:**
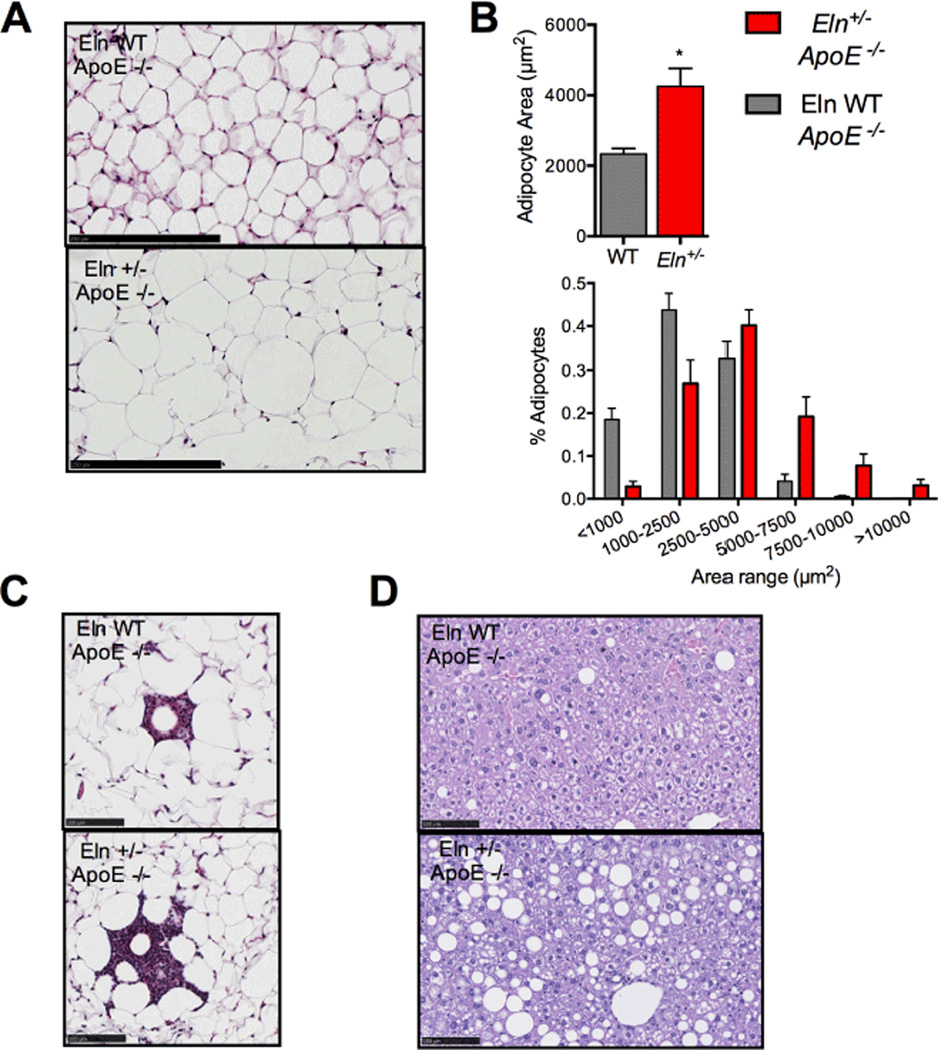
Adipocyte hypertrophy and lipid accumulation in *Eln*^+/−^ *ApoE*^−/−^ tissue. A: Masson’s trichrome staining of tissue representative of 7 sections each of epWAT from Eln WT and *Eln*^+/−^ mice on *ApoE*^−/−^ background fed control chow (scale bar 250 µm). B: Quantification of hypertrophy of epWAT in *Eln*^+/−^ tissue on *ApoE*^−/−^ background and control chow observed in A (mean ± SEM; n = 7). C: Differential levels of inflammation surrounding lipid droplets in Masson’s trichrome stained epWAT from Eln WT and *Eln*^+/−^ mice on *ApoE*^−/−^ background fed control chow (scale bar 100µm). D: Accumulation of lipid droplets in representative hematoxylin-eosin stained liver sections from Eln WT and *Eln*^+/−^ mice on *ApoE*^−/−^ background fed control chow (scale bar 100µm). *P≤ 0.05.
